# Polarization response and scaling law of chirality for a nanofibre optical interface

**DOI:** 10.1038/s41598-017-17133-3

**Published:** 2017-12-06

**Authors:** Mark Sadgrove, Masakazu Sugawara, Yasuyoshi Mitsumori, Keiichi Edamatsu

**Affiliations:** Research Institute of Electrical Communication, Tohoku University, Sendai, 980-8577 Japan

## Abstract

Two port optical devices couple light to either port dependent on the input photon state. An important class of two-port devices is that of evanescently-coupled interfaces where chirality of photon coupling can lead to important technological applications. Here, we perform a fundamental characterization of such an interface, reconstructing the two-port polarization response over the surface of the Poincaré sphere for an optical nanofibre. From this result, we derive a chirality measure which is universal, obeying a one parameter scaling law independent of the exact parameters of the nanofibre and wavelength of light. Additionally, we note that the polarization response differs qualitatively for single and multiple coupled emitters, with possible implications for sensing and the characterization of waveguide coupled spins.

## Introduction

Among recent progress in optical interfaces, the use of evanescent coupling to micro and nanoscale optical devices is important due to the chirality of coupling in these cases and its implications for next generation quantum optics applications^1^. The simplest interfaces which exhibit chirality are those with two output ports, a class which includes nanobeam waveguides, optical nanofibres, photonic crystal waveguides, and appropriately coupled whispering gallery resonators. Applications include waveguide based quantum optics^2–8^, nano-optical isolators^9^, single photon mirrors^10^, and coupling of light with both mechanical^11,12^ and magnonic^13,14^ excitations in matter. Additionally, such two-port interfaces may be combined to produce multi-port devices such as circulators^15^. In all of these cases, chirality arises from the spin-orbit interaction of light^16,17^. The directionality of coupling allowed by these devices gives rise to potential applications, chief among them the possibility of coupling distant spins *deterministically* ^1^ with applications to quantum networks^18^.

Formally, we may treat such evanescently coupled light-matter interfaces as two port devices where output intensity is a function of photon polarization at the interface. We refer to the dependence of the output port intensity on the polarization state as the *polarization response function* (PRF) of the interface. The PRF for each output port exists on the surface of the Poincaré sphere which parameterizes the polarization state by angles *θ* and *ϕ*. The PRF is a fundamental property of the interface similar to the transfer function associated with general linear systems, and can be defined for any polarization sensitive optical element. Nonetheless, although seminal demonstrations of chirality for interfaces in nanophotonics have recently been made^19^, to the best of our knowledge, the complete PRF for a light-matter interface with chiral coupling has never been measured.

As a related matter, the question of whether the PRF of a given interface exhibits *universality* is of importance. A simple example of universality in optics is that of an ideal polarizing beam splitter (PBS). Universality in this context is the useful property that an arbitrary (ideal) PBS has the same polarization response for any wavelength of light. A more general definition of universality is found in fields such as solid state physics^20,21^ and, more recently, cold atom physics^22,23^ where universality is ascribed to any property of a system which obeys a single-parameter scaling law. Previous investigations^10,19,24,25^ suggest that the chirality of coupling to light-matter interfaces is not universal, because it depends on the proportion of the mode which is evanescent, a property strongly dependent on the device dimensions and the light wavenumber.

Here, we experimentally measure the complete polarization response ${\mathcal I}$ of a specific, two-port nano-optical interface namely a point scatterer coupled to the fundamental modes of an optical nanofibre (ONF). We reconstruct the PRF for this system over the entire surface of the Poincaré sphere, and show that this allows a particularly elegant interpretation of chirality in terms of rotation and counter-rotation of the PRF. Somewhat surprisingly, we also find that this chirality measure is technically a universal property in that it obeys a one-parameter scaling law with no dependence on the details of the fibre radius or light wavelength. This finding is enabled by our consideration and measurement of the PRF for samples with different fibre radii in contrast to other recent studies^10,19,24,25^. Finally, we discuss the use of the PRF to distinguish between single and multiple scatterers on an ONF surface and its potential application to the characterization of systems of multiple emitters coupled to a single nanowaveguide.

## Results

**Figure 1:**
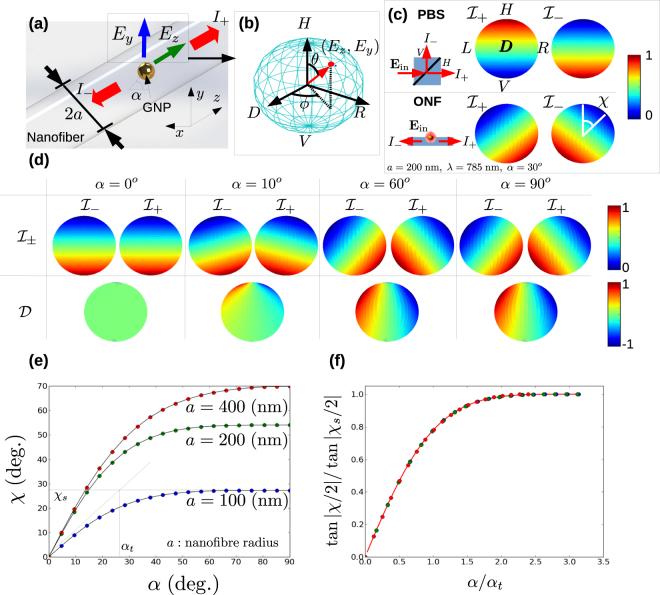
Theoretical polarization response of coupling to an ONF (**a**) Depiction of a gold nanosphere on the surface of an optical nanofibre. The azimuthal position of the particle is given by *α* and the excitation field has components *E*_*z*_ and *E*_*y*_. The intensity in the ±z direction is given by *I*_±_. (**b**) The polarization state of the scattered light is shown on the Poincaré sphere. (**c**) Comparison of PRFs for an ideal polarizing beam splitter (PBS) (upper panel) and for an ONF (lower panel) (radius *a* = 200 nm, nanosphere azimuthal position of 30 degrees, incident light wavelength 785 nm). States *H*, *V*, *L*, *R*, and *D* are as defined in the main text. The tilt angle of *I*___ away from the vertical is denoted by *χ*. (**d**) Table showing the variation of ${ {\mathcal I} }_{\pm }$ (first row) and ${\mathcal{D}}$ (second row) as a function of the azimuthal angle *α*. In all cases the orientation of the Poincaré sphere is the same as shown in (**c**). (**e**) The behavior of *χ* as a function of *α* for several different nanofibre radii *a* as indicated. The saturation value *χ*_*s*_ and the turning point *α*_*t*_ are indicated for the *a* = 100 nm case. (**f**) Data from (**e**) rescaled with the same data point colors used for the same nanofibre radii. The thick red line is the scaling function given by equation (1)

The principle of our experiment is illustrated in Fig. 1(a). Following a method similar to Petersen *et al*.^19^, we use a gold nanosphere (GNS) located at an azimuthal angle *α* on the nanofibre surface as an optical antenna to re-radiate input light from a polarization controlled source^19^. The GNS preserves the polarization of the input light, allowing us to realize an effective point dipole source with arbitrary polarization in the *y* – *z* plane. The PRF ${ {\mathcal I} }_{\pm }$ is given by the intensity of light coupled to the nanofibre ±*z* propagating fundamental modes as a function of **P**, the input polarization state which resides on the surface of the Poincaré sphere. We note that throughout the paper we consider only light coupled to the quasi-*y* polarized fundamental modes of the nanofibre. Experimentally this is guaranteed due to the *y* – *z* polarization plane of the input light and the single mode nature of the fibre from which the optical nanofibre is fabricated (See Methods). We neglect the light coupled to the vacuum modes because it is irrelevant to the characterization of the polarization response.

Figure 1(c) compares the theoretical polarization response function for an ideal polarizing beam splitter (PBS) and an optical nanofibre with parameters as shown. We use the labels *H* and *V* for light polarized parallel and perpendicular to the nanofibre axis (*z*-axis) respectively. Additionally, the right and left hand circular polarizations are denoted *L* and *R* respectively. Diagonal and antidiagonal polarizations are labelled *D* and *A* respectively. From Fig. 1(c), it may be seen that while the PRF for a PBS shows simple splitting between H and V components, for an ONF, the PRF components are generally rotated by an angle *χ* with respect to the vertical. Note that the point defined by $(\theta =\pm {\chi }^{o},{\varphi }={90}^{o})$ on the Poincaré sphere is the polarization for which coupling in the $\mp$ direction goes to zero. As the angle *χ* approaches 90°, the PRF of coupling to the nanofibre approaches that of a PBS, albeit with polarization splitting occuring along the *L* – *R* axis rather than *H* – *V*. This dependence on the handedness of the polarization is what leads to the “chiral” moniker for such interfaces. In the remainder of the paper, we will take the value *χ* as a measure of the chirality of the system.

Figure 1(d) is a table which shows how the theoretical PRF varies as *α* increases. In row one of the table, it can be seen that increasing *α* from 0 to *π*/2 results in rotation and counter rotation of ${ {\mathcal I} }_{+}$ and ${ {\mathcal I} }_{-}$ respectively. This is the origin of chirality for the nanofibre, which in general implies that ${ {\mathcal I} }_{\pm }$ differ for the same input polarization state. We can also define the directionality ${\mathcal{D}}=({ {\mathcal I} }_{+}-{ {\mathcal I} }_{-})/({ {\mathcal I} }_{+}+{ {\mathcal I} }_{-})$ ^19^. In row two of Fig. 1(d), we see that ${\mathcal{D}}$ also has a simple behavior on the Poincaré sphere, with lobes representing +*z* and −*z* directionality separating and rotating in opposite directions as *α* increases from zero. The exact behavior of the rotation *χ* as a function of *α* depends on the nanofibre radius as shown in Fig. 1(e). However, it turns out that the data can always be rescaled in such a way that it collapses onto a single curve. In order to reveal the scaling behavior, we consider tan|*χ*/2| rather than the angle *χ* itself. Then, it may be shown that the quantity tan|*χ*/2|/tan|*χ*_*s*_/2| lies on a single curve when plotted against the scaled azimuthal angle *η* = *α*/*α*_*t*_ as seen in Fig. 1(f). Here, the turning point *α*_*t*_ is rigorously defined as the value of *α* where the tangent intersects with the saturation value:

$${\frac{{\rm{d}}\chi }{{\rm{d}}\alpha }|}_{\alpha =0}\times {\alpha }_{t}={\chi }_{s}\mathrm{.}$$

It can be shown that the curve on which the scaled points lie - the *scaling function* - is given by

1 $$F(\eta )=2\frac{\mathrm{cosec}(\eta /\mathrm{2)}}{{\mathrm{cosec}}^{2}(\eta \mathrm{/2)}+1},$$

where *η* = *α*/*α*_*t*_. (A complete derivation of the scaling function is given in the supplementary material.) In this sense, the chirality as defined by *χ* may be said to be a universal property since it obeys a scaling law which does not depend on the parameters of the ONF or of the light used. Physically, the quantity tan(*χ*/2) is equal to the ratio of the magnitudes of the longitudinal and transverse components of the ONF fundamental mode. (See supplementary material). This quantity is intuitively a measure of chirality of the fundamental mode with maximum chirality being theoretically achieved when the two components have equal magnitude.

**Figure 2:**
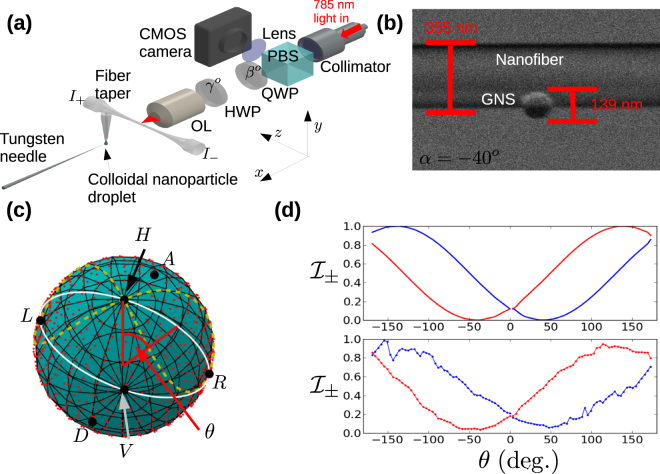
Experimental setup. (**a**) The experimental setup is shown. OL denotes objective lens, HWP half wave plate, QWP quarter wave plate and PBS polarizing beam splitter. The camera uses a complementary metal-oxide semiconductor (CMOS) sensor. (**b**) SEM image of a gold nanosphere on an optical nanofibre. The nanofibre diameter is 2*a* = 355 nm and the sphere diameter is 139 nm. The azimuthal position of the sphere on the nanofibre is *α* = −40°. (**c**) Points sampled in the experiment are shown on the surface of the Poincaré sphere (red dots). Small black circles mark the position of each of the six principle polarization states as indicated. The dashed, yellow line shows the trajectory taken on the Poincaré sphere for the data presented by Petersen *et al*.^19^. (**d**) Thoretical prediction (top panel) and experimental measurements (bottom panel) for the sample shown in (**b**) and the trajectory on the Poincaré sphere shown by a white line in (**c**). In both cases the blue line corresponds to ${ {\mathcal I} }_{+}$ while the red line corresponds to ${ {\mathcal I} }_{-}$.

We now turn to the experimental measurement of the phenomena discussed above. We used the system shown in Fig. 2(a) to deposit and illuminate GNSs on the surface of a nanofibre as seen in Fig. 2(b). By using both a quarter wave plate (QWP) and a half wave plate (HWP) in the optical beam path we can control the illumination light polarization (and thus the polarization of light scattered by the GNS) over the entire Poincaré sphere. To evaluate ${\mathcal I}$ experimentally, we first measured the two-port intensity *I*_±_(*β, γ*) on a regular grid of quarter-wave plate angles *β* and half-wave plate angles *γ*. Because the input state to the waveplates is fixed by the PBS to be ${{\bf{P}}}_{{\rm{i}}n}={\mathrm{[1,}\mathrm{0]}}^{T}$, (where ${\mathrm{[1,}\mathrm{0]}}^{T}$ is the Jones vector corresponding to horizontal polarization), we can calculate the polarization state at each pair of wave plate angles on the grid giving

$${\bf{P}}={\hat{M}}_{{\rm{HWP}}}(\gamma ){\hat{M}}_{{\rm{QWP}}}(\beta ){{\bf{P}}}_{{\rm{i}}n},$$

where ${\hat{M}}_{{\rm{HWP}}}$ is the Jones matrix for a half wave retarder and ${\hat{M}}_{{\rm{QWP}}}$ is that for a quarter wave retarder. Finally, we reconstruct the PRF on the Poincaré sphere using the mapping

2 $$\theta =2{\tan }^{-1}(|{P}_{y}/{P}_{z}|),\,{\varphi }={\rm{\arg }}({P}_{z})-arg({P}_{y}),\,{ {\mathcal I} }_{\pm }(\theta ,{\varphi })={I}_{\pm }(\beta ,\gamma ),$$

where *P*_*z*_ and *P*_*y*_ are the complex horizontal and vertical components of the polarization state respectively and arg(*c*) denotes the angle of the complex number *c* in the complex plane. We can also define ${\rm{\Delta }}=({I}_{+}-{I}_{-})/({I}_{+}+{I}_{-})$ ^19^. We can transform Δ into the directionality ${\mathcal{D}}$ using the mapping

$$\theta =2{\tan }^{-1}(|{P}_{y}/{P}_{z}|),\,{\varphi }={\rm{\arg }}({P}_{z})-{\rm{\arg }}({P}_{y}),\,{\mathcal{D}}(\theta ,{\varphi })={\rm{\Delta }}(\beta ,\gamma \mathrm{).}$$

The theoretical PRF can be calculated from *I*_±_ as detailed in the methods.

**Figure 3:**
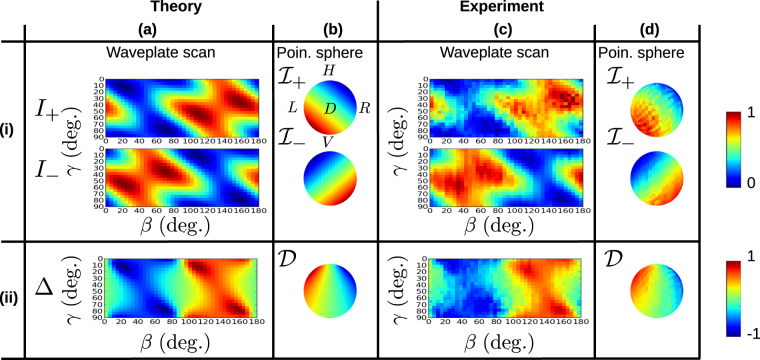
Table of theoretical and experimental results. Column (**a**) row (i) shows *I*_±_ as functions of the waveplate angles as predicted by theory. (**b**(ii)) The associated PRF ${ {\mathcal I} }_{\pm }$ plotted on the surface of the Poincaré sphere. Note that all Poincaré sphere plots in this figure have the same orientation as shown in the upper plot here. (**c**(i)). Experimentally measured *I*_±_ for the sample shown in Fig. 2(b),(d(i)). Reconstructed PRF ${ {\mathcal I} }_{\pm }$ plotted on the surface of the Poincaré sphere. (**a**(ii)). The theoretically predicted directionality Δ as a function of the wave plate angles. (**b**(ii)) ${\mathcal{D}}$ on the surface of the Poincaré sphere. (**c**(ii)) Experimentally measured value of Δ, as derived from the data in row (i). (**d**(ii)) The reconstructed value of D on the surface of the Poincaré sphere.

Optical measurement of the polarization response was performed by focusing a polarization controlled light beam (wavelength *λ* = 785 nm) on the surface of the nanofibre and scanning the *z*-axis to locate the GNS by detecting light scattered into the nanofibre guided modes. Once located, we used a computer controlled system to rotate the waveplate angles in a sequence synchronized with the detection of photons from the ±*z* ends of the nanofibre using avalanche photo-detectors (APDs). For 40 different QWP angles between 0° and 180°, we scanned the HWP angle in 20 equal increments between 0° and 90°. The 800 points which are sampled by this method are shown on the surface of the Poincaré sphere in Fig. 2(c). A one dimensional slice through the total scan, as indicated by the white line in Fig. 2(c), is shown in Fig. 2(d) for both theory (upper panel) and experiment(lower panel). In both cases, chirality is clearly evident in the different behavior of *I*_+_ (blue line) compared with *I*_−_ (red line). Specifically, it may be seen that *I*_+_ corresponds to the reflection of *I*_−_ in the line *θ* = 0. This behavior corresponds to the rotation of ${ {\mathcal I} }_{\pm }$ in opposite directions as seen in Fig. 1(d). Figure 3 shows a comparison of theoretical predictions and experimental measurements performed on the sample shown in Fig. 2(b). Columns (a) and (b) of row (i) in Fig. 3 show the predicted waveplate scan data *I*_±_ and the associated PRF ${ {\mathcal I} }_{\pm }$ respectively. We can immediately see the value of the full characterization of the polarization response on the Poincaré sphere. The patterns of minima and maxima whose meaning is unclear in the waveplate scans of Fig. 3(a(i)). are found to constitute a simple rotation of the non-chiral PRF as seen in Fig. 3(b).(i). Our experimental results shown in Fig. 3(c(i)) and (d(i)). display good qualitative and quantitative agreement with the theory. In particular, the experimental waveplate scan data is seen to correspond to the expected rotated structure of ${ {\mathcal I} }_{\pm }$ on the Poincaré sphere. We find similarly good correspondence of the directionality in row (ii) of Fig. 3. Again, the associated behavior of ${\mathcal{D}}$ on the Poincaré sphere agrees with the theoretical prediction. Our results demonstrate that a complete charaterization of the PRF can lead to a simplified understanding of the chirality of a nano-photonic interface.

To proceed, we note that the tilt angle *χ* of the PRF on the Poincaré sphere is a natural way to characterize the chirality of the sample. However, in general, *χ* has a behavior which is dependent on both *ka* and the azimuthal angle *α*. In particular, as seen in Fig. 1(e), both the saturation value *χ*_*s*_ and the turning point *α*_*t*_ at which growth in *χ* saturates, depend on the nanofibre radius. Nonetheless, as we demonstrated earlier, a scaling function exists for the behavior of *χ*. We now apply the scaling function as a method of comparing chirality for different samples.

**Figure 4:**
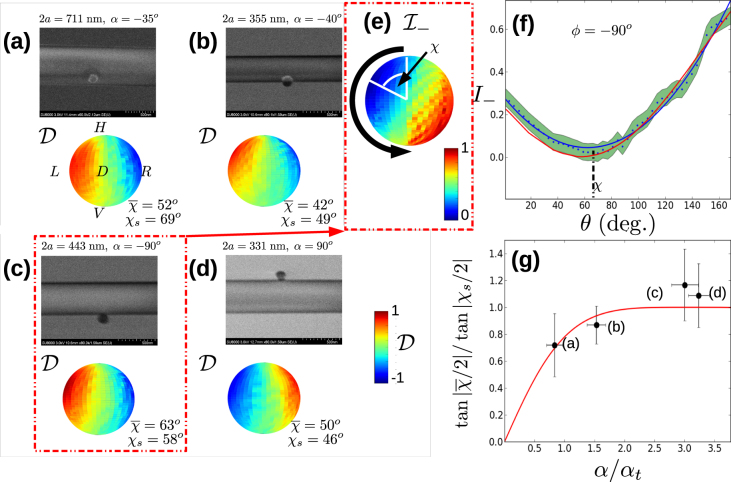
Comparison of results using the scaling function. (**a**–**d**) Four experimentally obtained results with parameters as shown. In each case, the upper panel shows a scanning electron microscope image showing the optical nanofibre with radius *a* and gold nanoparticle at azimuthal angle *α* as shown. The average angle of chirality $\overline{\chi }$ is shown in each case. Note that in all cases the Poincaré sphere orientation is the same as shown in (**a**). (**e**) *I*_*_*_ for the case shown in (**c**). The thick black arrow indicates the trajectory taken to produce the 1D slice shown in (**f**). The value of *χ* for this case is indicated. (**f**) 1D slice through the data shown in (**e**) used to extract the value of *χ* as shown. Blue dots show experimentally measured values, while the green region shows ±1 standard deviation for the data. The red line shows the theoretical prediction while the blue line shows a quadratic fit to the data to guide the eye. (**g**) Scaled data (discrete points) corresponding to each data set shown in (**a**–**d**) as indicated. Vertical error bars show the standard deviation over the four values of *χ* averaged to produce $\overline{\chi }$. Horizontal error bars show the range of the systematic error due to the uncertainty in the value of *α* (±4°). The solid red curve shows the scaling function *F*.

Figure 4(a–d) show SEM images of four samples (upper panel in each case) along with their measured directionalities. Note that the sample shown in Fig. 4(b) is the same as that for Fig. 2(b). We analysed the rotation *χ* of ${ {\mathcal I} }_{\pm }$ for each sample using the method illustrated in Fig. 4(e) and (f). Specifically, we took a one dimensional slice through the data indicated by the thick black line in Fig. 4(e) to give the experimental data shown by blue points in Fig. 4(f). The green shaded region in this figure shows ±1 standard deviation over 5 measured values. The red line in Fig. 4(f) shows the theoretical prediction for this data and the blue line shows a quadratic fit to the data to guide the eye. The value of *θ* at which the minimum occurs is *χ*. We performed this analysis for both ${ {\mathcal I} }_{+}$ and ${ {\mathcal I} }_{-}$ and for data where the particle was on the front side and the back side of the nanofibre to give a total of four data points for each sample. In principle, the absolute value of *χ* in each case should be the same. However, the random error in ascertaining the minimum in each case along with small misalignments of the beam axis relative to the nanofibre axis lead to different values in practice. By averaging the data we can reduce the effects of such random and systematic errors.

We denote the averaged value by $\overline{{\chi }}$. To produce the scaled data, we calculate the quantity $\tan \,|\overline{\chi }\mathrm{/2|/}\,\tan \,|{\chi }_{s}\mathrm{/2|}$. (See Methods for more information.) The scaled data, is shown in Fig. 4(g) plotted against $\eta =\alpha /{\alpha }_{t}$. The error bars show the standard deviation of the four values averaged to give $\overline{\chi }$. It may be seen that the scaled values agree with the scaling function *F*(*η*) (red line in Fig. 4(g)) within experimental error. The scaling function thus gives us a meaningful way to compare chirality across the different nanofibre diameters and particle positions seen in Fig. 4(a–d). Another way to appreciate the usefulness of the scaling function is to note that although Maxwell’s equations guarantee the same results for constant $2\pi a/\lambda =ka$ due to their scale invariance properties, if *ka* changes, in general one must recompute the solution. However, in the case of chirality as characterized by *χ*, scaling using the experimentally measurable parameters *χ*_*s*_ and *α*_*t*_ allows us to compare data with a single, one parameter function *F*(*η*) *for any k and a*. This scaling property therefore goes beyond the standard scale-invariance of Maxwell’s equations. Given the existence of this scaling behavior, we come to the somewhat surprising conclusion that optical nanofibres can also be universal devices in the sense that their polarization response is independent of the detailed system parameters other than input light polarization.

Finally, we discuss a possible application of the PRF with respect to distinguishing between single and multiple scatterers on the nanofibre surface. In the case where the scatterer is a quantum emitter, it is possible to ascertain whether one or many emitters is present within a given illuminated area by measuring the intensity correlation function ${g}^{\mathrm{(2)}}(\tau )$ and testing for anti-bunching at delay time *τ* = 0. However, for classical scatterers, this test is not possible. Furthermore, while estimates of the total number of scatterers on the fibre have been be made using absorption spectroscopy techniques^19^, determining how many scatterers are present within the illumination region is not possible with this technique.

The PRF provides a method for testing the presence of a single classical scatterer as follows: For single scatterers the PRF always has a minimum which lies on the great circle passing through states *L*, *H*, *R* and *V* on the Poincaré sphere. That is, the *ϕ* coordinate of the polarization state which gives minimal coupling is 90°. This fact is seen in Fig. 4(a)–(d), where the minimum of the PRF is located close to *ϕ* = 90° in each case.

Physically, this occurs due to the fact that the mode function of the nanofibre is always of the form $\varepsilon ={\varepsilon }_{y}{{\bf{e}}}_{y}+{\rm{i}}{\varepsilon }_{z}{{\bf{e}}}_{z}$ for real ${\varepsilon }_{y}$ and ${\varepsilon }_{z}$, where ${{\bf{e}}}_{y}$ and ${{\bf{e}}}_{z}$ are the unit vectors along the *y* and *z* axes respectively. Zero coupling occurs when the polarizaiton of the scattered light is orthogonal to that of the mode function, so the incident field in this case has the form ${E}_{y}{{\bf{e}}}_{y}-{\rm{i}}{E}_{z}{{\bf{e}}}_{z}$ for real *E*_*y*_ and *E*_*z*_. Applying the definition of *ϕ* from equation (2), it may be seen that these simple elliptical polarization states all reside on the aforementioned great circle of the Poincaré sphere.

This is *not* the case in general for situations where two (or more) scatterers are present in the illumination region on the nanofibre’s surface. Neglecting nanofibre mediated dipole-dipole interactions between the two scatterers, in this case considerable rotation about the *H* – *V* axis is possible compared to the single particle case because the minimum of intensity coupling is now caused by interference in the guided modes between the light coupled at each scatterer rather than orthogonality of the guided mode and the scattered light.

The angle *ϕ* at which the PRF minimum is found therefore provides evidence for the presence of either single or multiple emitters being present in the illumination region. We have observed such rotations about the *H* – *V* axis in the multi-scatterer case as we will report elesewhere. This property may have applications to particle sensing and to the characterization of coherently excited multiple emitters coupled to the same waveguide, for example, rudimentary quantum cascaded systems and networks. It also remains to be calculated how dipole-dipole interactions between emitters mediated by the fibre guided modes could be detected using the PRF.

## Discussion

A principle attraction of the polarization response function as measured here is the simplification of the understanding of chirality which it provides. Previous measurements of the coupled intensity as a function of waveplate angle show a detailed dependence which, while clearly demonstrative of chirality, is by no means easy to interpret^19^. Here, our complete characterization allows for the simple observation that the polarization response function in the case of chiral coupling is just a rotated version of the non-chiral PRF. Additionally, it is possible to meaninfully compare the ONF as a two-port, polarization sensitive device with any other such devices such as an ideal PBS. Thus, measurement of the complete PRF for coupling to the nanofibre allows a deeper understanding of the chiral coupling phenomenon.

Understanding is further enhanced by our demonstration that the chirality as characterized by *χ* also obeys a scaling function. In optics, the most well-known example of scaling is the scale-invariance of Maxwell’s equations themselves. In that case, a solution of Maxwell’s equations for a given wavenumber *k* for a structure with linear dimension characterized by *a* will be valid for a different system with the same product *ka*, all other things being equal. The scaling we have demonstrated here removes even this dependence on *ka*, with only the single parameter *α* needed to characterize the behavior of scaled data. That is, there is no need to compute the PRF for arbitrary combinations of ONF radii and wavelengths. Rather, since the scaling law is universal, the behavior of the suitably scaled function tan(*χ*/2) is always governed by the function *F*(*η*) for *any* value of *ka*.

Because the concepts of scaling and universality are not commonly invoked in optics, we briefly discuss some practical objections which might be raised as to the usefulness of the scaling results presented here. First, it might be pointed out that while technically universality exists, practically nanowaveguide cross-section dimensions are chosen to be of order *λ*/2 to maximize coupling. While this is a fair observation, we note that by using a resonator (of which chiral varieties exist), selective coupling into the fundamental mode can be greatly enhanced by the Purcell effect without particular sensitivity to the waveguide dimensions. This means that in principle, the choice of diameter is not fixed by the need to maximize coupling. Second, it might be objected that the results given here are specific to cylindrical waveguide geometries, making the concept of universality restricted. Although the present scaling function applies specifically to the case of ONFs, the idea of the PRF is completely general, and it is reasonable to expect scaling of chirality to exist for other waveguide geometries. The feature which gives rise to the scaling behavior is the smooth increase of chirality as we move from a position of minimum intensity and tangential field boundary conditions to a position of maximum intensity with the field normal to the waveguide surface. Such features are common for fundamental modes for any waveguide geometry, making it likely that scaling will also be present in other geometries.

The PRF is a fundamental property of an evanescently coupled interface, but it may also have practical applications. In particular in the case of multiple scatterers coupled to the same nanowaveguide (for example in cascaded quantum systems and rudimentary quantum networks), characterization of the complete PRF is necessary to find the polarization state which gives maximal coupling to the waveguide. It may also help to confirm the existence of single classical scatterers coupled to a waveguide - something that until now was only possible for quantum emitters using correlation methods. For these reasons, we believe that characterization of the PRF will be an important tool for understanding many next generation chiral optical interfaces.

## Methods

### Sample preparation

Tapered optical fibres were produced in-house from commercial single mode optical fibres (cutoff wavelength = 730 nm) using a standard heat and pull technique. The waist region of the tapers had a length of ≈1 mm over which the diameter was approximately constant. In this region (referred to as the *nanofibre* region) GNSs were deposited using a tungsten needle which passed through a colloidal solution of GNSs and touched the nanofibre surface. We passed 630 nm wavelength light through the nanofibre and imaged light scattered in the nanofibre region on the CMOS camera. When deposition of a GNS ocurred, high intensity scattering was observed at the point where the needle had touched the nanofibre after the needle itself had been retracted. The success rate of producing a single GNS when a deposition occurred was approximately 50%.

### Optical measurement

The prepared sample was mounted on an automated *z* – *y* stage and moved to overlap with the focus of the laser spot formed by the 10x microscope objective. The laser light came from a free-running laser diode with a center wavelength of 785 nm. The sample’s *x*– axis position was deliberately moved away from the exact focal point in order to produce a larger effective spot size. This reduced the amount of noise and intensity drift due to vibrations and other movements of the nanofibre. First, we performed optical measurements sweeping the focal spot over the nanofibre axis for the bare nanofibre as a control. Nanofibres which showed any scattering peaks at this stage (e.g. due to impurities on the nanofibre surface) were rejected. If this initial test was passed, we introduced GNSs to the nanofibre surface as discussed in the Sample Preparation section above. GNSs were detected optically by large amplitude, localized peaks in the scattering rate into the nanofibre as measured using APDs. The peak signal to background ratio was typically of order 100∼1000. Due to the prohibitive time required to take multiple data sets at each point on the Poincaré sphere, we took data five times for just one sweep where the incident light polarization was set to be linear. The mean error (standard deviation) found for this data was assumed to be representitive of the mean error for all data points taken. For all samples, polarization characterization was performed twice - the second time with the nanofibre flipped 180° about the nanofibre axis relative to the first time.

### Scanning electron microscope (SEM) measurement

After optical data acquisition had been completed, we analysed each sample using a SEM. Gold nanoparticles were identified by their unique shape and size (diameter ≈150 nm). We associated gold nanoparticles located in SEM images with optical measurements using two principle methods. First, by depositing several nanoparticles on one nanofibre at well-defined separations, we could correlate inter-particle distances measured using the SEM with those found using optical measurements. The distances between particles as measured optically and using the SEM typically agreed to within 5 *μ*m. Second, because of the good agreement found between theory and experiment, we could use comparison between theoretical predictions for SEM measured parameters and actual optical measurements as a way to identify specific particles detected using the SEM with specific optical measurements.

### Data processing

Plots as a function of HWP and QWP angles are raw data normalized by max(max(*I*_+_)-min(*I*_+_), max(*I*_-_)-min(*I*_-_)). Because  the minimum value is less than 3% of  the maximum value in all cases we measured, this value is approximately the global maximum of the data over both *I*_+_ and *I*_−_. Plots of Δ as a function of HWP and QWP angles are derived from this normalized data according to the formula ${\rm{\Delta }}=({I}_{+}-{I}_{-})/({I}_{+}+{I}_{-})$. The polarization response function shown on the surface of the Poincaré sphere is derived from the normalized data by nearest neighbor interpolation to produce an evenly-spaced data set on the *θ* – *ϕ* grid. For the scaled data shown in Fig. 4(g), the data points are the average value of *χ* measured for *I*_±_ for the particle on the front side of the nanofibre, and the back side of the nanofibre as achieved by flipping the sample 180° about the nanofibre axis. This averaging procedure cancels systematic errors produced by small misalignments between the nanofibre and the illuminating beam. The scaling of $\overline{\chi }$ was performed by dividing through by the value $\tan \,|{\chi }_{s}\mathrm{/2|}$, where *χ*_*s*_ was found by solving the nanofibre eigenvalue equation for the HE_11_ mode for a nanofibre with a radius given by the value measured using the SEM. The value of *α*_t_ was found in the same way.

### Theoretical polarization response function calculation

In order to derive a theoretical expression for the PRF, we first calculate the intensity at each port as a function of the waveplate angles. This is done by assuming the GNS can be treated as a point dipole emitter with an induced dipole ${\bf{d}}=p{\bf{E}}$, where *p* is the scalar polarizability of the GNS, and ${\bf{E}}={E}_{y}{{\bf{e}}}_{y}+{E}_{z}{{\bf{e}}}_{z}$ is the input light field with complex components *E*_*y*_ and *E*_*z*_. The intensity is then found to be^19^

$${I}_{\pm }\propto |{{\boldsymbol{\varepsilon }}}_{\pm }\cdot {\bf{E}}{|}^{2},$$

where · is understood to indicate the Hermitian inner product. The quantities **ε**_±_ are the ±*z* propagating, quasi-*y* polarized HE_11_ modes of the optical nanofibre^26^. Coupling to the quasi-*x* polarized HE_11_ modes is negligible for the cases studied here, and does not contribute to the chiral response.

Note that in practice, we normalize *I*_±_ so that their maximum values are 1. Additionally, we note that our analysis is focused on the polarization response and does not consider the channeling efficiency of scattered light into the nanofibre guided modes. This has been measured elsewhere and has a polarization averaged value of ∼20% for the parameters considered here^27^.

### Data availability

Data is available on request.

## Electronic supplementary material

Supplementary information
