# Supplementary material for: Polarization response and scaling law of chirality for a nanofibre optical interface
Source: Sci Rep. 2017 Dec 6;7:17085. doi: 10.1038/s41598-017-17133-3 (PMC5719059; doi:10.1038/s41598-017-17133-3)
Supplement: Supplementary file 1 — Supplementary information [file 41598_2017_17133_MOESM1_ESM.pdf]

Supplementary material for "Polarization response and scaling law of chirality for a nanofibre optical interface"

Mark Sadgrove, Masakazu Sugawara, Yasuyoshi Mitsumori, Keiichi Edamatsu[1]

[1] *Research Institute of Electrical Communication, Tohoku University, Sendai 980-8577, Japan*

I. DERIVATION OF THE SCALING FUNCTION FOR CHIRALITY

Here we present a derivation of the scaling function $F(\eta)$ used in the paper. First, we present the definitions to be used in the derivation of the scaling function. Fig. 1(a) shows the system being considered. A gold nanoparticle is situated on the surface of an optical nanofibre at an azimuthal angle α. It is illuminated by an external optical field with components E_y and E_z which are in general complex. As depicted in Fig. 1(b), the values of the field components can be controlled over the entire Poincaré sphere, in the basis H, V, where H is the horizontal (z) polarization component and V is the vertical (y) polarization component. The polarization response functions $\mathcal{I}_{\pm}$ give the relative intensity of light in $\pm z$ directions as a function of polarization state as shown for $\mathcal{I}_{+}$ in Fig. 1(c). In general, the polarization response function is a rotated version of the $\alpha = 0$ polarization response with a minimum located at an angle χ as measured from the north pole (i.e. the H state).

In what follows we will take the tilt angle χ of the polarization response function on the Poincaré sphere as our chirality measure. $\chi = 0^o$ implies no chirality and $\chi = 90^o$ implies maximum chirality. Maximum chirality can only be achieved for perfect circular polarization of the guided mode which in principle can never occur, although increasing the diameter and the refractive index of the waveguide both bring the maximum chirality closer to $\pi/2$. In general, a larger value of χ implies that a directionality of ± 1 can be achieved closer to the polarization for maximum coupling efficiency to the nanofibre.

A. The behavior of χ

Fig. 1(d) shows a table of polarization response functions plotted on the Poincaré sphere for different a and α. Notice that the saturation chirality χ_s increases as the nanofibre diameter increases. (Of course, the coupling to the nanofibre fundamental mode tends to zero as the radius increases, so this increase is of limited practical use.)

Fig. 2(a) shows the chirality χ as a function of α for each nanofibre radius in Fig. 1(d). The values of χ are computed by numerically finding the value of θ on the Poincaré sphere which gives the minimum of the polarization response function. Saturation behavior is clearly visible, and it is notable that apart from having different saturation values χ_s, the curves for each value of the radius also show different turning points α_t at which growth behavior rolls over to saturation.

B. Connection between χ and the nanofibre mode polarization

The value of χ corresponds to the angle θ on the Poincaré sphere which gives the minimum coupling. At this angle, the excitation light polarization is orthogonal to the nanofibre fundamental mode polarization at the azimuthal position α at which the scatterer is situated. Consideration of two extreme cases helps to determine the exact relationship. First, consider when $\alpha = 0$. In this case, the fundamental mode has no longitudinal component implying that $\chi = 0^o$ and the mode polarization at this position is V corresponding to $\theta = 180^o$. On the other hand, when $\alpha = 90^o$, the mode polarization reaches its maximum ellipticity corresponding to maximum chirality. The absolute upper limit is $\chi = 90^o$ which would correspond to perfectly circularly polarized light, implying a state R, or $\theta = 90^o$ on the Poincaré sphere. From these two examples, it is clear that the relationship between chirality and the mode polarization θ coordinate is

$$180^o - \chi = \theta. \tag{1}$$

This is useful, because the mode polarization angle can be calculated analytically, eliminating numerical errors associated with detecting χ from the polarization response function on the Poincaré sphere. The solid lines in Fig. 2(b) show the values of χ calculated directly from the nanofibre mode function Poincaré sphere angle θ. Precisely, the lines show the value

$$\chi = 180^o - 2\tan^{-1}(|E_y|/|E_z|). \tag{2}$$

C. The scaling function

1. *Scaling of variables*

We will now move on to show that a scaling function exists for the chirality χ. Until now, we have used angles in degrees for easy visualization. In what comes, we will use radians as appropriate for mathematical manipulations.

As Fig. 2(b) and Eq. 2 show, the key to understanding the behavior of χ lies in the nanofibre fundamental mode components E_y and E_z. We will restrict ourselves to

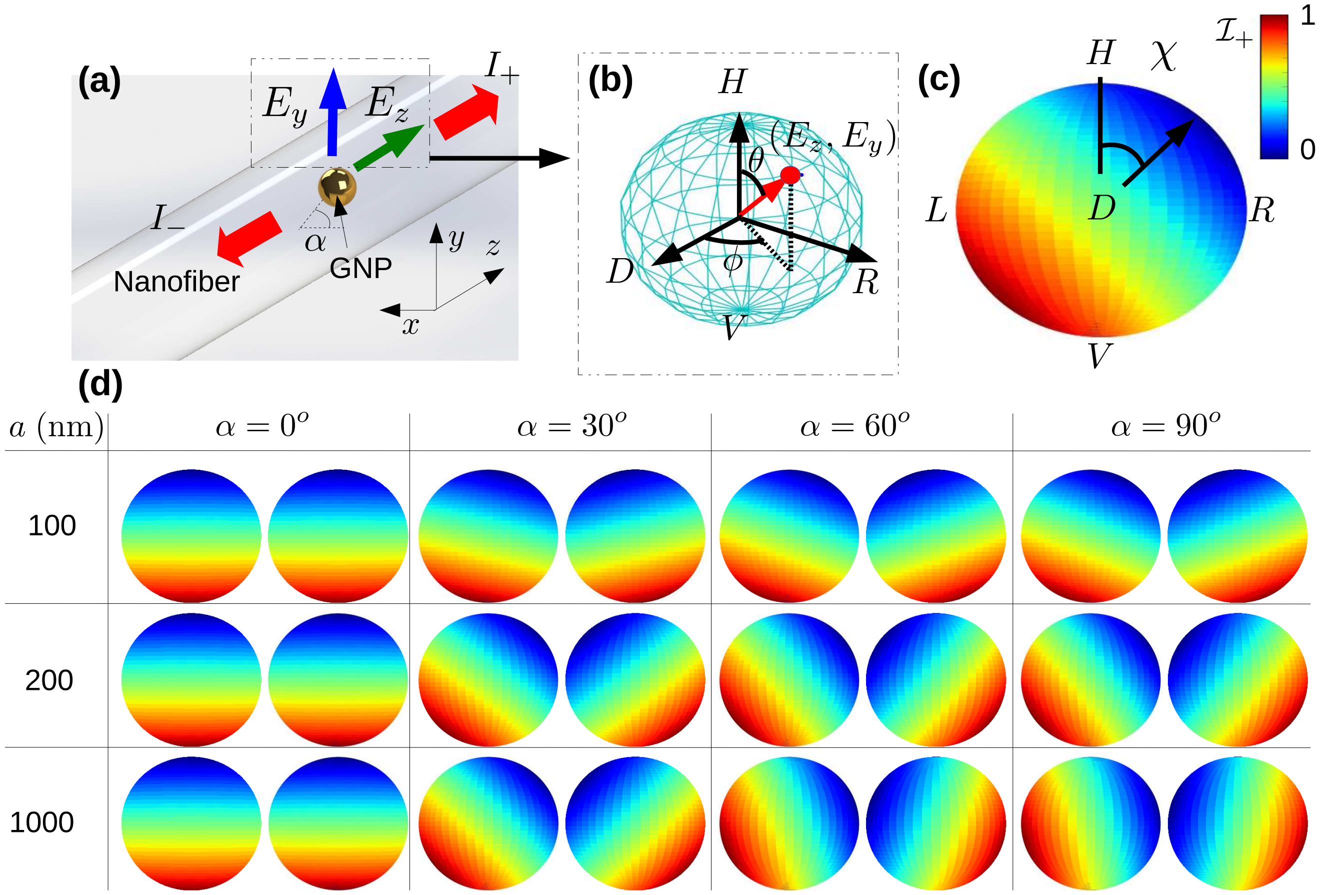

FIG. 1. (a) Concept of the experiment: a gold nanosphere on the surface of a nanofibre at an azimuthal angle α is excited by an external field with components E_y and E_z. The values of the field components can be controlled to span the surface of the entire Poincaré sphere,as depicted in (b). For the purposes of investigating chirality, it is most instructive to project onto the plane containing states H and R. (c) shows the polarization response functions $\mathcal{I}_+$ for such a projection, with polarization states H (horizontal $= z$), V (vertical$=y$), L (left hand circular), R (right hand circular) and D (diagonal) shown. Due to the chirality of coupling, the polarization response function tilts on the Poincaré sphere by an angle χ. (d) is a table showing how the rotation of the polarization response function rotates as a function of α for different nanofibre radii a with the same axes as shown in (c).

evaluating the mode functions at the nanofibre surface. The nanofibre mode function for the quasi $y-$polarized HE_{11} fundamental mode of a nanofibre is given by [1]

$$E_y = \mathrm{i}A\beta a\frac{J_1(u)}{wK_1(w)}\left[\left(\frac{1-s}{2}K_0(w) - \frac{1+s}{2}K_2(w)\right)\cos^2\alpha + \left(\frac{1-s}{2}K_0(w) + \frac{1+s}{2}K_2(w)\right)\sin^2\alpha\right] \quad (3)$$

$$E_z = -AJ_1(u)\sin(\alpha), \quad (4)$$

where $u^2 + w^2 = v^2$, $v = ka\sqrt{n_{\text{co}}^2 - n_{\text{cl}}^2}$, $k = 2\pi/\lambda$, $s = (1/u^2 + 1/w^2)/(J_n'(u)/uJ_n(u) + K_n'(u)/wK_n(w))$ and J_n and K_n are Bessel functions of the first kind and modified Bessel functions of the second kind respectively.

At this point, it would typically be mentioned that u and w must be found by solving the eigenvalue equation for the nanofibre fundamental mode which in general depends on the product of the wave number of the mode k and the nanofibre radius a. However, in the present work, we are deriving a scaling function which has *no dependence* on the specific parameters of the nanofibre system. We therefore know that the dependence on w and u must drop out during the derivation, otherwise such scaling behavior could not exist. It is therefore possible to forgo the usual definition of the eigenvalue equation and leave the exact values of u and w undefined.

Proceeding, with a little algebra, we find

$$\left|\frac{E_y}{Ez}\right| = \frac{\beta a}{wK_1(w)}\left[\frac{1-s}{2}K_0(w)\text{cosec}\alpha + \frac{1+s}{2}K_2(w)(2\sin\alpha - \text{cosec}\alpha)\right], \quad (5)$$

where $\text{cosec}\alpha = 1/\sin\alpha$ is the co-secant function. Because we have $E_y > 0$ but $E_z \geq 0$, it will make sense for us to consider the inverse of Eq.5 in what follows, since

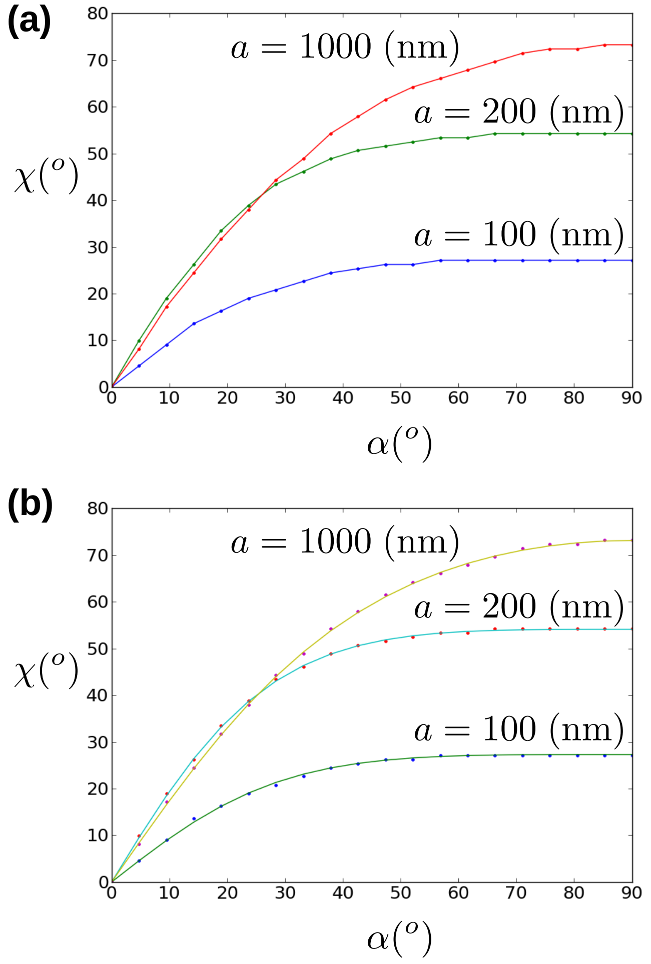

FIG. 2. (a) Variation of χ with α for nanofibre radii a as indicated. The values of χ were extracted by numerically finding the minimum value of the coupling function $\mathcal{I}_+$ on the Poincaré sphere. (b) Same as (a), but with solid lines showing the value of χ calculated directly from the nanofibre mode function.

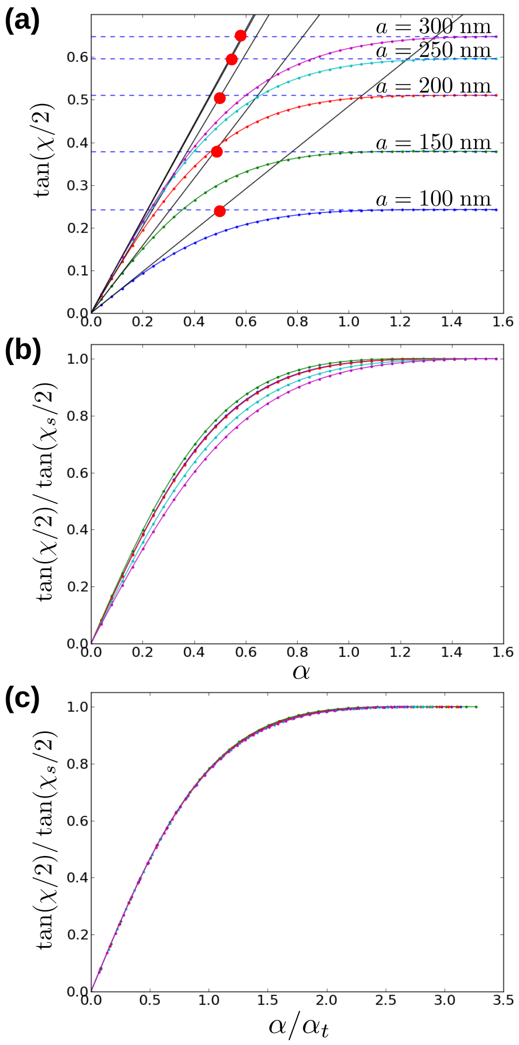

FIG. 3. (a) Values of $\tan(\chi/2)$ as a function of α for nanofibre radii as indicated. Dashed blue lines show the saturation level in each case. Black lines show the tangent line to each curve at $\alpha = 0$. Red dots mark the intercept of each tangent line with the saturation value. The value of α at which the intercept occurs is labeled α_t.(b) Same as (a), but with scaling of each curve by $1/\tan(\chi_s/2)$ for the appropriate value of χ_s in each case. (c) Same as in (b), but with additional scaling of α by the factor $1/\alpha_t$

it is well behaved for all values of E_y and E_z. It can be shown from Eq. 2 and standard trigonometric identities that

$$\tan(\chi/2) = \left|\frac{E_z}{E_y}\right|. \tag{6}$$

Figure 3(a) shows $|E_z/E_y|$ as a function of α for five different nanofibre radii. The behavior is seen to be qualitatively the same as that for χ vs. α. In each case, the tangent to the curve at $\alpha = 0$ is shown as a black line and the saturation level is shown as a dashed blue line. The point where the saturation level and the tangent line intercept defines the turning point of the curve. The α value at which this occurs is denoted α_t.

We can arrive at an analytical formula for α_t as follows: first the saturation level is found by inserting $\alpha = \pi/2$ into Eq. 5 giving

$$\left|\frac{E_z}{E_y}\right|_{\alpha=\pi/2} = \frac{wK_1(w)}{\beta a\left[(1-s)/2K_0(w) - (1+s)/2K_2(w)\right]}. \tag{7}$$

Next, we note that when $\alpha \approx 0$ we have

$$\left|\frac{E_z}{E_y}\right|_{\alpha\approx 0} = \frac{wK_1(w)\alpha}{\beta a\left[(1-s)/2K_0(w) + (1+s)/2K_2(w)\right]}. \tag{8}$$

The value of α_t is then found by equating Eqs. 7 and 8 and solving for α giving

$$\alpha_t = \frac{(1-s)/2K_0(w) - (1+s)/2K_2(w)}{(1-s)/2K_0(w) + (1+s)/2K_2(w)}. \tag{9}$$

We now come to the scaling of variables. First, we perform a simple amplitude scaling to bring the saturation values of all curves to unity. To do this, we simply divide by the factor $\tan(\chi_s/2)$. As shown in Fig. 3(b), this produces curves which saturate at unity, but which do not otherwise overlap.

However, if we go further and scale α by the factor $1/\alpha_t$, we see that all the curves now lie on top of each other to an excellent approximation (Fig. 3(c)). We refer to this behavior as a scaling law for the chirality of an optical nanofibre, or, alternatively, we say that the quantity χ is governed by a one parameter scaling law.

2. *Derivation of the scaling function formula*

The above demonstrations have made a convincing case that a scaling law exists for χ. However, without a functional form for the one parameter scaling law, it is difficult to quantify how well the scaling law holds or to check the scaling law with experimental data. Therefore, we now proceed to derive the functional form of the curve which the scaled points lie on.

Assigning the scaling variable η the value

$$\eta = \alpha/\alpha_t, \tag{10}$$

and the scaling function $F(\chi)$ the form

$$F(\chi(\alpha)) = \tan(\chi(\alpha)/2)/\tan(\chi_s/2), \tag{11}$$

we note that the scaling function we seek is the union of all the coordinates $(\eta, F(\chi(\alpha)))$. To proceed, we would like to express F as a function of η. This is simple: we just substitute $\alpha = \alpha_t\eta$, subject to $0 \le \eta \le \pi/2\alpha_t$, into F. Using Eqs. 5 and 7, we can write

$$F(\eta) = \frac{\frac{1-s}{2}K_0(w) + \frac{1+s}{2}K_2(w)}{\frac{1-s}{2}K_0(w)\mathrm{cosec}(\alpha_t\eta) + \frac{1+s}{2}K_2(w)(2\sin(\alpha_t\eta) - \mathrm{cosec}(\alpha_t\eta))} \tag{12}$$

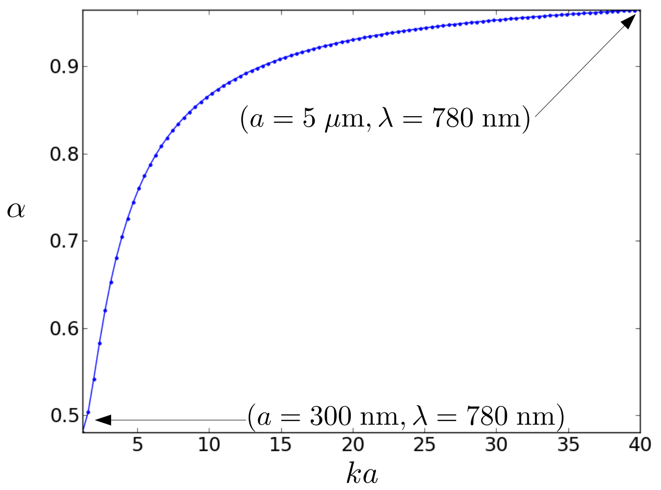

FIG. 4. The dependence of α on ka is shown.

The terms in w are preventing us from finding a scaling law parameterized by η alone. We will make a series of manipulations to reduce Eq. 12 to a function of α_t and η alone, before finally arriving at a single parameter scaling law in terms of η. First, we define $\Gamma = [(1-s)/2]K_0(w) + [(1+s)/2]K_2(w)$ for convenience. We note that

$$\alpha_t = ([(1-s)/2]K_0(w) - [(1+s)/2]K_2(w))/\Gamma. \tag{13}$$

We now perform the following manipulation of Eq. 12: multiply through by $1 = (1/\Gamma)/(1/\Gamma) \times \mathrm{cosec}(\alpha_t\eta)/\mathrm{cosec}(\alpha_t\eta)$. This transforms Eq. 12 to the equivalent form

$$F(\eta) = \frac{\mathrm{cosec}(\alpha_t\eta)}{\alpha_t\mathrm{cosec}^2(\alpha_t\eta) + \frac{1}{\Gamma}(1+s)K_2(w)}. \tag{14}$$

Now, from Eq. 13, we find that

$$\alpha_t\Gamma = \frac{1-s}{2}K_0(w) - \frac{1+s}{2}K_2(w). \tag{15}$$

Adding $((1+s)/2)K_2(w)$ to both sides and gathering terms, we find

$$1 - \alpha_t = \frac{1}{\Gamma}(1+s)K_2(w). \tag{16}$$

This allows us to write $F(\eta)$ parameterized only by α_t:

$$F(\eta) = \frac{\mathrm{cosec}(\alpha_t\eta)}{\alpha_t\mathrm{cosec}^2(\alpha_t\eta) - \alpha_t + 1}. \tag{17}$$

So far all our manipulations have been completely general. However, the hypothesized scaling law holds for any physically valid value of α_t. We are therefore free to judiciously choose α_t in deriving a form for $F(\eta)$. As shown in Fig. 4, for a silica, vacuum clad nanofibre, α lies between 0.5 and 0.97 to a good approximation. Due to the restriction $0 \le \eta \le \pi/(2\alpha_t)$, choosing α_t as small as possible will allow us to define $F(\eta)$ over the widest possible domain. For this reason, $\alpha_t = 0.5$ is a good choice. Substituting into Eq. 14, we find the following one parameter scaling law:

$$F(\eta) = 2\frac{\mathrm{cosec}(\eta/2)}{\mathrm{cosec}^2(\eta/2) + 1}. \tag{18}$$

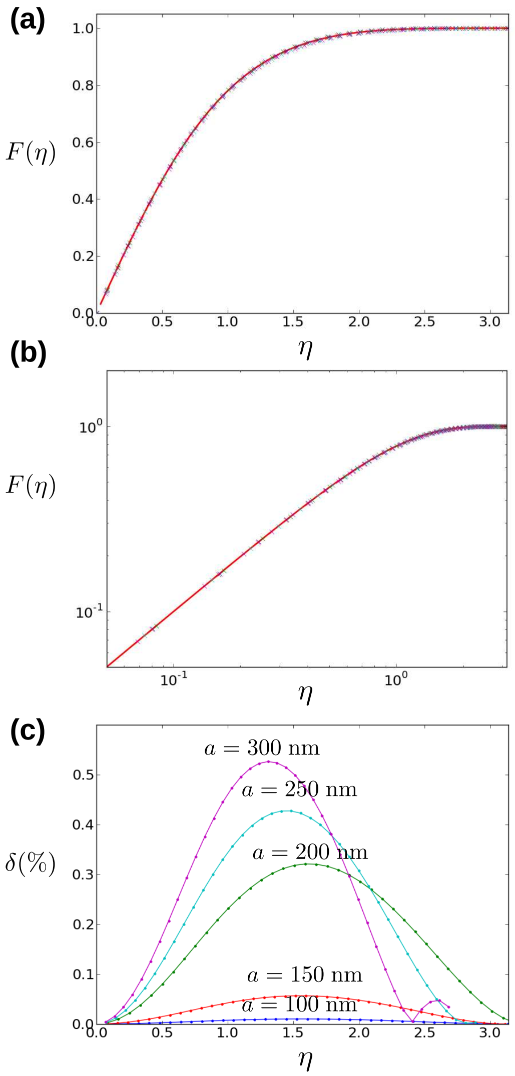

FIG. 5. (a)The scaling function $F(\eta)$ is shown along with the data from Fig. 3 (shown as individual points). (b) Log scale version of (a). (c) The error δ for each data point from Fig. 3 is shown for nanofibre radii as indicated.

Figure 5(a) and (b) show the scaling function Eq. 18 (red line) compared with the scaled values of $\tan(\chi/2)$ shown in Fig. 3 on linear and logarithmic scales respectively. Qualitatively, the fit may be seen to be excellent

More quantitatively, the difference between the true values of $\tan(\chi/2)$ and $F(\eta)$ is given by

$$\delta = |\tan(\chi/2)/\tan(\chi_s/2) - F(\eta)| \qquad (19)$$

as shown in Fig. 5(c). The error is seen to be systematic, but at worst, the peak error is under 0.6% for the parameters investigated here. This is below the error seen in numerical estimations of χ from reconstructions of the polarization response function on the Poincaré sphere, let alone the experimental error which is of order 10%. Therefore, the scaling function represents a sufficiently accurate and very convenient way to compare experimental measurements of chirality with theory.

[1] "Fundamentals of optical waveguides", K. Okamoto. Academic Press (2000).
